# Comparative Analysis of Object Digitization Techniques Applied to the Characterization of Deformed Materials in Ballistic Tests

**DOI:** 10.3390/s20185017

**Published:** 2020-09-04

**Authors:** Filipe Dmengeon Pedreiro Balbino, Khrissy Aracélly Reis Medeiros, Carlos Roberto Hall Barbosa

**Affiliations:** 1 Postgraduate Programme in Metrology, Pontifical Catholic University of Rio de Janeiro, Marquês de São Vicente Street, 225, Gávea, Rio de Janeiro 22451-900, Brazil; filipedmengeon@hotmail.com; 2 Mechanical Engineering Department (DEM), Optical Fiber Sensors Laboratory (LSFO), Pontifical Catholic University of Rio de Janeiro, Marquês de São Vicente Street, 225, Gávea, Rio de Janeiro 22451-900, Brazil; kmedeiros@puc-rio.br

**Keywords:** digitization, ballistic tests, depth measurement, three-dimensional reconstruction, three-dimensional scanners

## Abstract

Several three-dimensional scanning methods have been developed and improved over the past 40 years. The peculiarities of each technique, associated with the computational advances of the period, allowed the increasing application and diffusion of the technology in several sectors, among them those related to metrology in ballistics and the testing of protective materials. The specific goal of such ballistic tests is to estimate the depth of indentation caused by projectiles. In particular, this study presents a comparative analysis between two three-dimensional optical scanning methods, taking into account the same object of interest. The comparative analysis was based on reference planes detected by Random Sample Consensus methodology in each cloud. By comparing the results of the different techniques, it was found for this case that three-dimensional reconstruction by stereo images estimated values closer to the real ones in comparison to those estimated by the structured light scanner, mainly due to the fact that, for three-dimensional reconstruction, the image acquisition was conducted statically.

## 1. Introduction

From the 21st century onwards, the advancement of technology and its accessibility in economic terms made three-dimensional (3D) digitization a powerful tool. Industry and society, in general, started to have systems available for digitizing physical objects and other structures with a high geometric fidelity, based on blue/white light technologies incorporated into high-resolution optics to reproduce parts details with precision in a short time. Three-dimensional scanning instruments have evolved with touch probes added to the optical system, constituting hybrid systems that can be used to acquire larger amounts of data faster than conventional touch systems, enabling metrological applications with increasingly smaller levels of measurement uncertainty [1], which explains its recent dissemination, allowing even dimensional analyses with a high level of reliability [2].

Since then, several areas have benefited from the emergence and subsequent popularization of the aforementioned new technologies. However, their application has not been widespread in the field of body armor testing, mainly due to the belief that 3D scanning technologies are associated with equipment and software with high acquisition costs and maintenance prices. In this context, the mechanical resistance of the material used in ballistic vests and their behavior under the impact of ammunition are conventionally assessed by measuring the depths of trauma observed in the plasticines clay.

Traditionally, calipers and similar instruments are used to measure trauma in body armor tests, because such measuring instruments meet the need for a resolution equal to or better than 1 mm, as required by the US National Institute of Justice (NIJ) standards NIJ 0101.04 [3] and NIJ 01.01.06 [4]. The caliper remains the most applied instrument in this area; however, when considering the number of necessary intermediate steps so that the trauma depth can be measured with this instrument, it yields a large uncertainty of the measured value. Additionally, considering that the measurements performed in the traditional way are highly dependent on the operator, the repeatability and reproducibility of the process may be compromised [5].

Thus, to solve this cost-related question, try to mitigate these process-related metrological problems, and provide satisfactory results for the application under discussion, surface digitization techniques were considered to reduce the number of intermediate steps and to establish some absolute benchmark against which the trauma can be measured. It is notable that conventional digitization techniques are a fertile field for applications of this type [6], added to the fact that such technology proves to be more advantageous regarding the metrological reliability of the data that it can provide [7].

Examples of conventional scanning techniques include those that use 3D scanners, such as the FARO 3D Objects Scanner [8], widely used in digitizing models and for acquiring spatial information about objects—as well as the 3D reconstruction technique using stereo images. The 3D reconstruction by stereo images is the most viable technique from the economic and implementation points of view, and is commonly applied in areas related to computer vision [9]. In addition, the 3D reconstruction technique by stereo images constitutes an important part of other more complex techniques, such as the Digital Image Correlation (DIC), whose digitization process is carried out in successive moments, making it possible to map the evolution of the object of interest over time [7].

As a result of the digitization of an object carried out by the aforementioned equipment, the three-dimensional coordinates of the points that compose that object are obtained. This set of points is called a point cloud, the composition of which usually varies from thousands to billions of points [10] and digitally represents the object of interest, with a greater or lesser degree of accuracy, depending on the equipment and technique employed [11]. Based on the data collected, the generated dense point clouds can be processed by a variety of software (Geomagic™, Polyworks™, Verisurf™, among others [12]) and used with the CAD (Computer-Aided Design) graphics software, which enables the three-dimensional reconstruction of the digitized information and even allows reverse engineering jobs [13,14].

In this sense, considering that the use of digitization technologies is not widespread in the area of body armor testing, this study aims at comparatively analyzing two techniques of the acquisition of shapes without contact applied to the characterization of deformed materials in ballistic tests. Particularly, the potential of two 3D image reconstruction techniques were assessed by comparing the point clouds generated by the techniques of structured light projection and 3D stereo image reconstruction using Charged Coupled Device (CCD) cameras.

The arrangement of this paper is as follows: Section 2 presents a short literature review regarding 3D scanning techniques; the theoretical principles of the body armor tests are given in Section 3; Section 4 presents the 3D scanning techniques used in this work; the materials and methods for the point cloud acquisition are presented in Section 5; the results and discussions are in Section 6; and the conclusions of the manuscript, including future works recommendations, are described in Section 7.

## 2. Review of Surface 3D Scanning

Given the wide variety of digitization techniques available, optimal results can be achieved with the application of digitization techniques and appropriate instruments for different applications, such as the survey of geodesic and forest canopy information [15]; the preservation of historical heritage [16,17]; anthropometric measurements [18]; and applications related to medicine, where it is possible to digitize organs and structures using computed tomography and magnetic resonance, allowing subsequent manipulation by doctors and surgeons to plan and execute surgical procedures [9].

Sa et al. [19] presented a comprehensive classification of the methods available for digitizing 3D shapes. In general, the methods were divided into contact and non-contact methods. Optical techniques are classified in the latter case, including those related to 3D Scanners and 3D Reconstruction by stereo images. As a precursor reference, Higgins’ work [20] stands out, which presents an algorithm that can digitally reconstruct an object based on two or more photographs of it. In this study, the author proposed a method to find the fundamental matrix *F* that relates the images’ points to those of the object, thus allowing its 3D reconstruction. The empirical foundations of this technique are based on human vision, where the brain, when fusing two images, allows the human being to have notions of depth and distance. Fundamentally, Higgins’ work [7,20] served as a basis for developing the 3D reconstruction techniques, photogrammetry, and DIC.

In 1983 and the following years, Sutton et al. [21] proposed a technique that made use of the results presented by Higgins which had at its core the “mapping” of the changes that occurred on the surface of a given object over time, allowing the analysis of displacements and deformations on a macro and microscopic scale (through the use of suitable lenses).

Regarding photogrammetry, it has become widely used as a digitization technique in geodetic sciences since the 2000s. Techniques such as Structure-From-Motion (SFM) [22] started to stand out in this scenario, mainly due to the low cost and the quality of the data set that can be obtained. The application of this technique, associated with lasers [23], the projection of light patterns [24,25], and inertial positioning systems [26], allowed the development of high-resolution 3D scanners capable of generating dense point clouds that are quite reliably faithful to the original objects.

In particular, applicability studies for 3D scanners and their comparison with conventional methods were developed by Asís et al. [27] with solar panels applications. Additionally, Xia et al. [28] made the comparison between conventional techniques and two different types of scanners, aiming at application in the textile area, while National Research Council Of The National Academies [5] studied it in the scope of the measurement of trauma depths in ballistic vest tests. The latter study was of high relevance for the area, since it served as the basis for several subsequent studies on the entire ballistic vest conformity assessment process [5].

## 3. Ballistic Tests on Bulletproof Vests (Body Armor)

There are currently several standards regarding the testing of ballistic vests, which vary according to the employment needs of each country or region, aiming to meet levels of protection related to certain ballistic threats. Currently the most used standardization is that formulated by the American National Institute of Justice, whose standards NIJ 0101.04 [3] and NIJ 01.01.06 [4] are widely known. For assessing the so-called “behind armor blunt trauma” in ballistic vests, standardized tests are carried out in multilayer shielding systems (MSS), using ammunition of specific calibers and speeds that comply with the aforementioned standards.

The aforementioned standards establish different levels of protection (Table 1), which vary according to the threat to which a specific ballistic vest or armor for individual use will be exposed, and also establish speed ranges for projectiles of each level.

The support material for ballistic tests consists of plastiline (a specific type of modeling clay), packed in a 610 × 610 × 197 mm container. Before conducting the test, the plastiline undergoes temperature conditioning, aiming to make its consistency similar to that specified in the NIJ 0101.04 [3] standard. According to this same standard, six shots are fired at the ballistic panel, with the deformation criteria being assessed in the first three traumas, where the depth should not exceed 44 mm [3]. In the other three shots, only the perforation criterion is observed—that is, confirming that there was no perforation of the ballistic panel.

**Figure 1:**
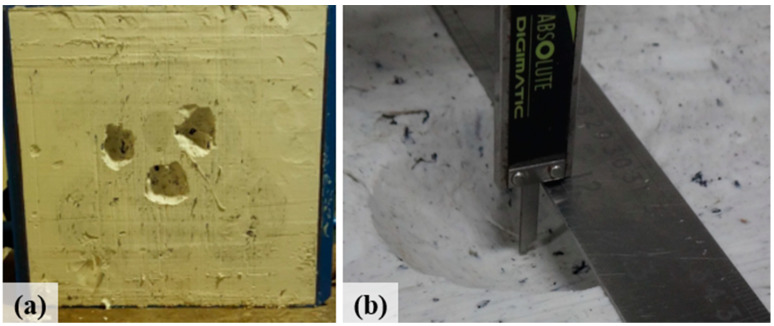
Original sample: (**a**) clay after “scraping”; (**b**) depth measurement with a caliper.

After receiving the shots, the modeling clay has the deformed aspect of Figure 1a and to measure the depths, a conventional caliper is commonly used for this application (Figure 1b).

The use of calipers was extensively addressed in [5], and some comparisons with coordinate measuring machines and 3D scanners were made. It was found that the caliper underestimates the depth measurements because it depends on the experience of the operator. Additionally, the research concluded that the combined uncertainty for the process using the caliper, taking into account the instrument and the operator, reaches 0.823 mm. The same measurement process performed with a measuring arm able to perform 3D scans led to an estimated uncertainty of 0.145 mm, a considerably superior result. Thus, considering that the caliper remains widely used in this context, the use of non-contact technologies has been widely proposed, as is the case of several surface digitization processes [29,30].

## 4. 3D Scanning Techniques

### 4.1. Optical 3D Scanners by Structured Light Projection

**Figure 2:**
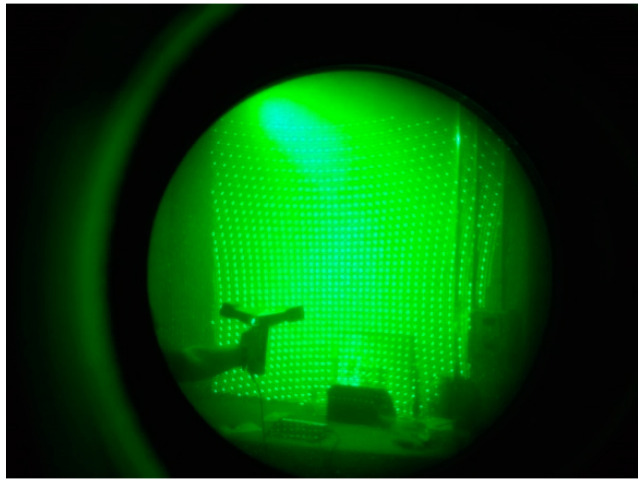
Infrared light dot projection [8] seen with the aid of a night vision camera.

In this type of equipment, the acquisition of information is conducted by two infrared cameras that capture a pattern also projected in infrared (Figure 2). Based on this information, 3D coordinates are calculated using triangulation. Smooth movements around the object are perceived by gyroscopes and accelerometers, and the information acquired by these sensors helps in the composition of the global coordinate system. A color camera also takes photos in conjunction with the infrared cameras, and its images serve to enhance the data collected and to add color information to the points [8].

For data acquisition to be carried out, in addition to the scanner, there is a need for software to perform the post-processing information and a computer with a good processing capacity to generate the final point cloud.

**Figure 3:**
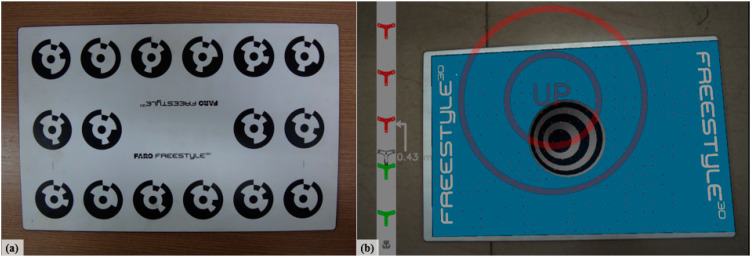
(**a**) Calibration plate; (**b**) calibration process displayed on the screen [8].

Initially, before any measurement is performed, the equipment needs to be calibrated (Figure 3). To mitigate the effects of local brightness, this procedure must be performed in the same place where the digitization is to be performed. Following the manufacturer’s guidelines (FARO^®^), the scanning must be made used at a distance between 0.3 and 0.8 m from the object, reaching a measurement uncertainty of 0.5 mm [31].

Structured light 3D scanners are devices that have the triangulation system as their working principle and have limitations regarding data acquisition, such as brightness and color, which may considerably affect the acquisition of information, as the projected pattern on the object can be attenuated or even absorbed by it.

In terms of operation, the distance between the scanner and the object is estimated by emitting short light pulses over it and measuring the time elapsed between the emission and the reception of the reflected light. It is emphasized that possible errors in the equipment’s time measurement can reduce the accuracy in the location of the digitized points on the surface [29].

### 4.2. 3D Reconstruction by Stereo Images

The use of stereo images for object reconstruction is a relatively simple technique to be used and aims to obtain the dimensional information of a given object based on at least a pair of images of that object.

The process for 3D reconstruction by stereo images is widely described in the literature [32,33]. Truco and Verri [34] present the process as one of the techniques applied in computer vision.

**Figure 4:**
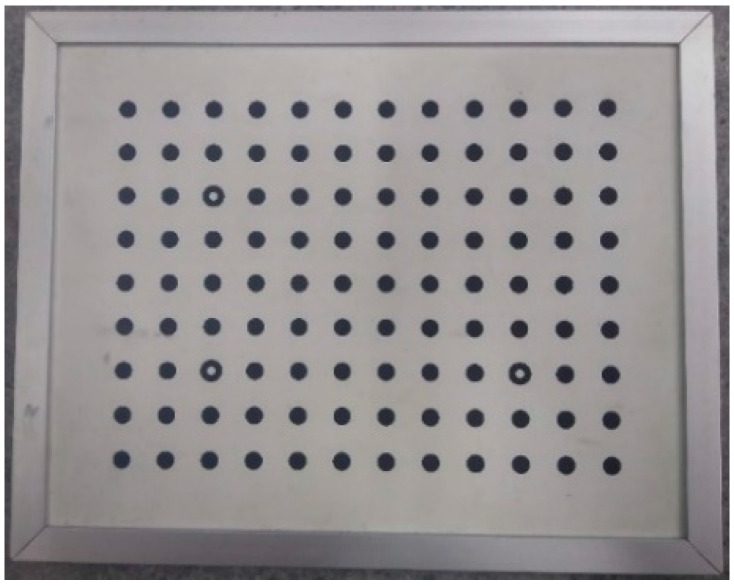
Plate used to calibrate the stereo camera system.

To obtain information of interest, the following sequence must be followed:

(a) Calibration of the cameras, which defines the internal and external parameters of the vision system used [34]. Calibration allows the acquisition to be carried out with some level of standardization of information at the level of the vision system employed, using some known reference standard (Figure 4).
(b) Acquisition of stereo images of the same object by a pair of identical or similar cameras. It should be noted that information such as the distance between cameras and other aspects, such as the luminosity and reflectivity of the surface of interest, can influence the acquisition process, as previously mentioned.
(c) A stereo analysis extracts information by comparing the two images and the location of objects in three-dimensional space [34].

## 5. Materials and Methods

**Figure 5:**
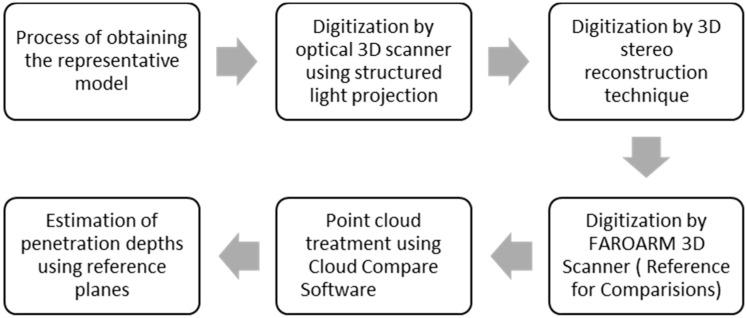
Flowchart of the structure of the subsections.

Aiming at comparing the two techniques of 3D reconstruction of objects described in the previous section, in the scope of application in ballistic vest tests, a digital model representative of the object of interest—the original sample—was used to carry out the comparative study. A flowchart with the sequential structure of the various processes employed in this work is shown in Figure 5, with each process being described in a separate subsection.

### 5.1. Process of Obtaining the Representative Model and Point Cloud

To compare the 3D digitizing techniques (Handheld 3D scanner [31], 3D reconstruction by stereo images [35], and FAROARM 3D Scanner [36]) in the present study, a standard sample was initially built on a wooden board using a 3D milling machine. This wooden sample reproduces on its surface the deformations observed in the modeling clay shown in Figure 1a and presents several advantages over the plastiline clay box originally used in the tests, such as ease of handling, portability, and the preservation of the machined shapes. Such factors make it more difficult to use the plastiline clay box as a sample to compare different techniques.

**Figure 6:**
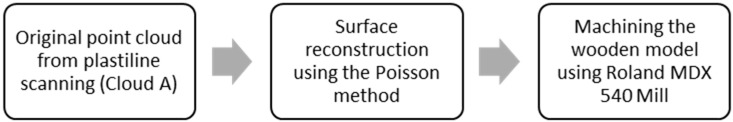
Flowchart of obtaining the wooden milled model.

The flowchart of Figure 6 depicts the process of obtaining the representative wooden model, which was later used to obtain the point clouds defined as Cloud B, Cloud C, and Cloud D.

The first digitization (Cloud A) refers to the traumas observed in the plastiline (original sample) after the impact on a particular ballistic vest. This first point cloud was obtained using the 3D FARO 3D Objects Scanner [8], with a reading time of approximately 30 s and a distance of approximately 50 cm from the object.

**Figure 7:**
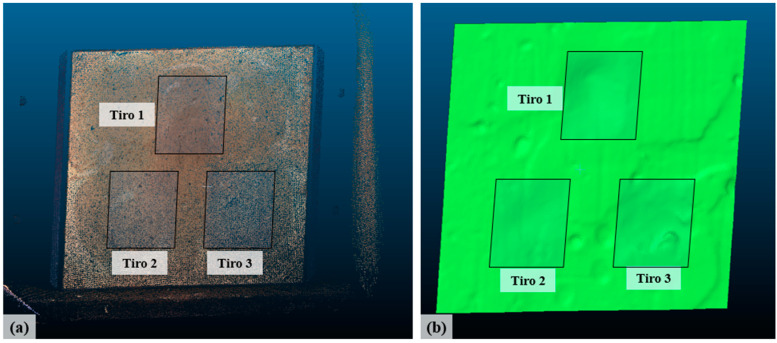
Digitization of plastiline (original sample): (**a**) cloud A and (**b**) the surface treatment, cleaning, and reconstruction of the region of interest using the Poisson method.

Based on this initial point cloud, properly treated with the aid of the Cloud Compare software [37], to eliminate points and unwanted artifacts present in the digitization, it was possible to obtain a cloud containing only the plastiline face deformed by the impacts (Figure 7a). A surface was then obtained (Figure 7b), using the Poisson surface reconstruction method [38].

**Figure 8:**
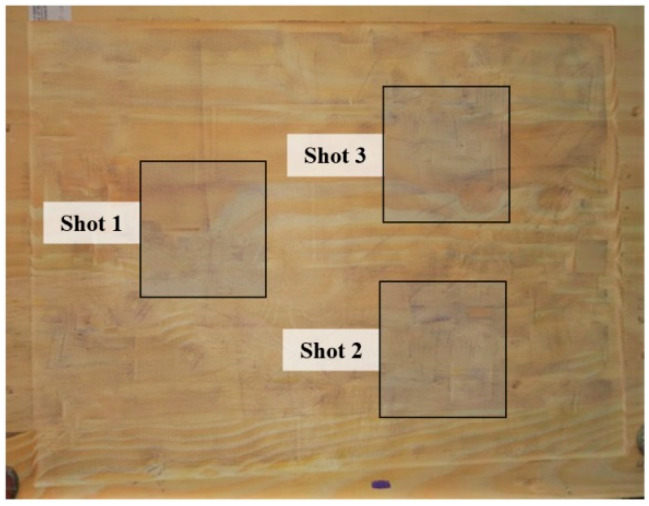
Milling: physical model made of wood.

Because of the aforementioned implications for the original sample, the object in Figure 7b was then used to make a physical model in wood (26.5 cm × 37.5 cm) using a Roland MDX-540 milling machine. Figure 8 shows the wooden model obtained. The use of this approach sought to obtain representativeness of the traumas that are usually observed in the execution of ballistic tests.

This manufactured physical model (MPM) was then subjected to three different digitization processes, as follows.

### 5.2. Digitization by the Optical 3D Handheld Scanner Using Structured Light Projection

**Figure 9:**
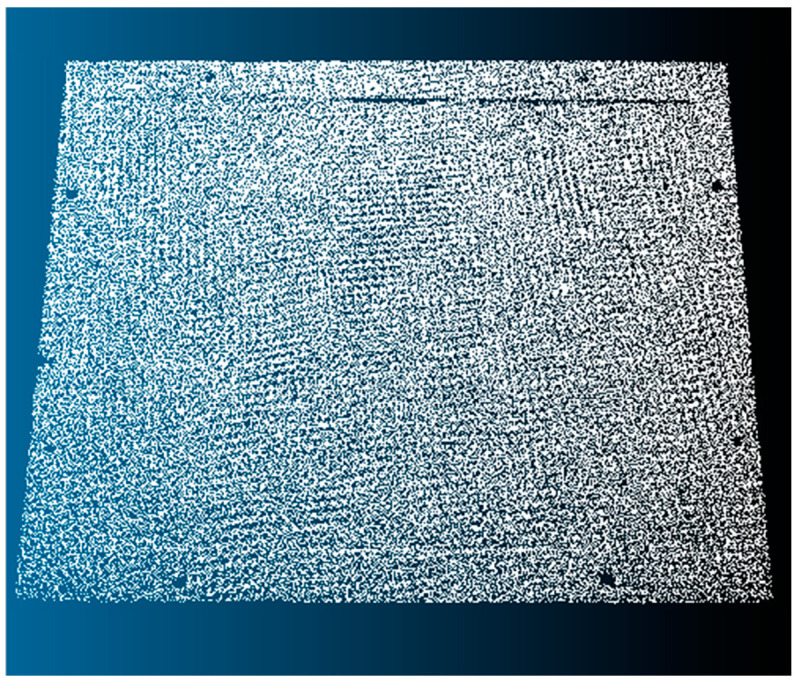
Point Cloud B, generated using the 3D Scanner FARO 3D Objects.

The wooden sample described in the previous section was first digitized using a structured light scanner manufactured by FARO, called the FARO 3D Objects 3D Scanner [31]. The scanner was previously calibrated using the manufacturer’s recommendations, and data acquisition was performed at the suggested optimal distance of 50 cm for 30 s. To generate the final point cloud, the FARO Capture and FARO Process software were used. As a result of this process, point cloud B shown in Figure 9 was obtained.

### 5.3. Digitization by 3D Stereo Reconstruction Technique

To digitize the wooden sample using the 3D reconstruction technique using stereo images, it was necessary to prepare the plate’s surface by dyeing it white and randomly inserting black dots on it (known as speckles). After that, it was possible to adjust and provide the 3D reconstruction software with the correct location of each surface region. In a real application, it is possible to project some light pattern on the clay box or add little black spheres and mix it with clay. The process of painting the plastiline surface proves to be unfeasible, both because of the oily nature of the modeling clay and because of the large amount of time spent in the process, compromising ballistic tests.

**Figure 10:**
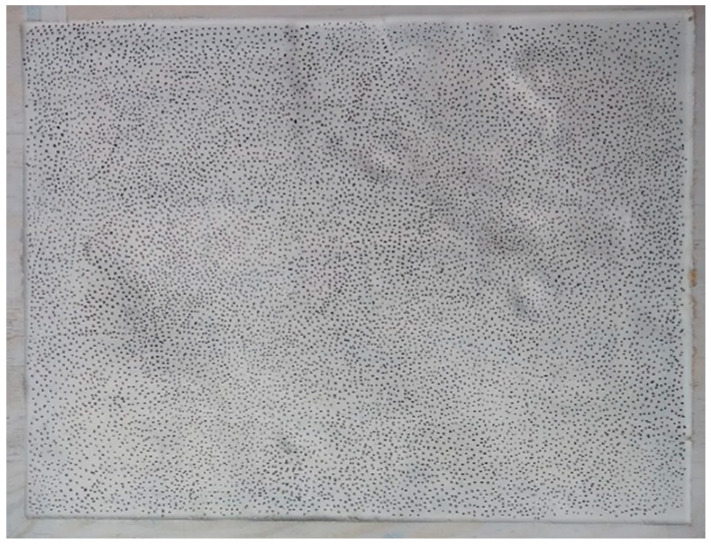
Physical model pigmented with aleatory black dots (speckles).

Once pigmented (Figure 10), the sample was then positioned, with the aid of fasteners, approximately 2 m from a pair of identical CCD cameras. The calibration of these cameras was made using a plate with holes with standardized distances to provide the correct information to the reconstruction software.

**Figure 11:**
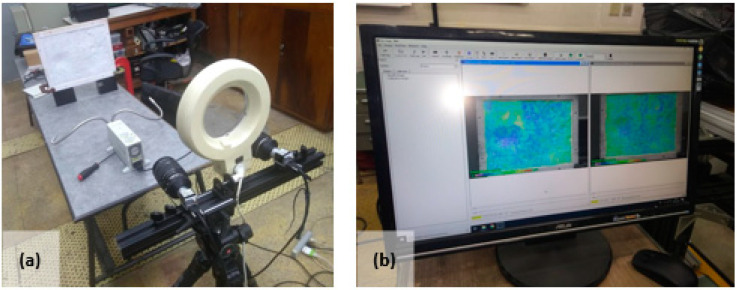
Stereo image capture process: (**a**) test setup for stereo image capture and (**b**) 3D reconstruction software.

Once the system was adjusted, the simultaneous capture of the images by the two CCD cameras is shown in Figure 11.

**Figure 12:**
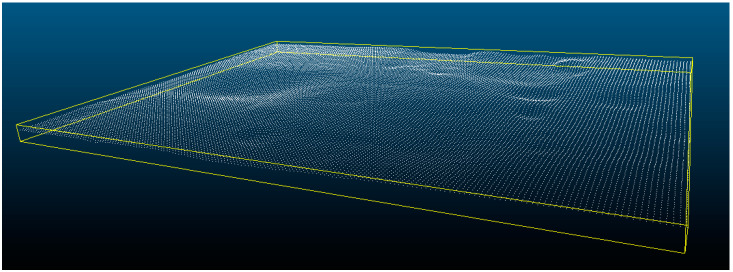
Point Cloud C from digitalization via 3D reconstruction by stereo images.

From the information collected, the point cloud representing the surface of the wooden sample could be estimated using the 3D reconstruction software, VIC 3D, from the company Correlated Solutions, making it possible to obtain Point Cloud C shown in Figure 12.

### 5.4. Digitization by FAROARM^®^ 3D Scanner.

**Figure 13:**
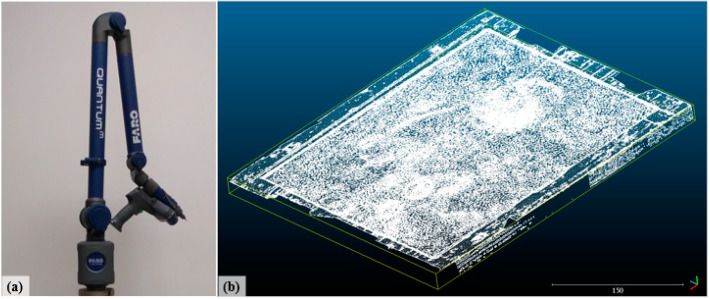
FAROARM 3D Scanner [36] (**a**) and the result of the machined physical model scanning process, (**b**) presented through the software CloudCompare.

This work aims to compare the results from two digitization techniques (structured light and 3D reconstruction). For this purpose, the results must be compared with some ground truth measurements. The FAROARM 3D Scanner (Figure 13) was selected for this purpose for the following reasons: availability, a measurement uncertainty of ±25 μm, the acquisition of a large number of points (2000 points per line with 40 μm of minimum spacing) [36] in a short period, and the possibility of scanning the whole machined physical model (MPM). The use of a Coordinate Measuring Machine (CMM) with a better accuracy would be more time consuming, and probably the deepest point on the surface would not be measured. The point cloud that originated from this process was named “Point Cloud D”.

### 5.5. Point Cloud Treatment via Cloud Compare Software

At the end of the data acquisition by different techniques, the next step consisted of processing and subsequent comparison between the three point clouds: Cloud B (digitization via structured light scanner) with Cloud D (obtained from the FAROARM 3D Scanner), and Cloud C (scanning via stereo images) with Cloud D.

**Figure 14:**
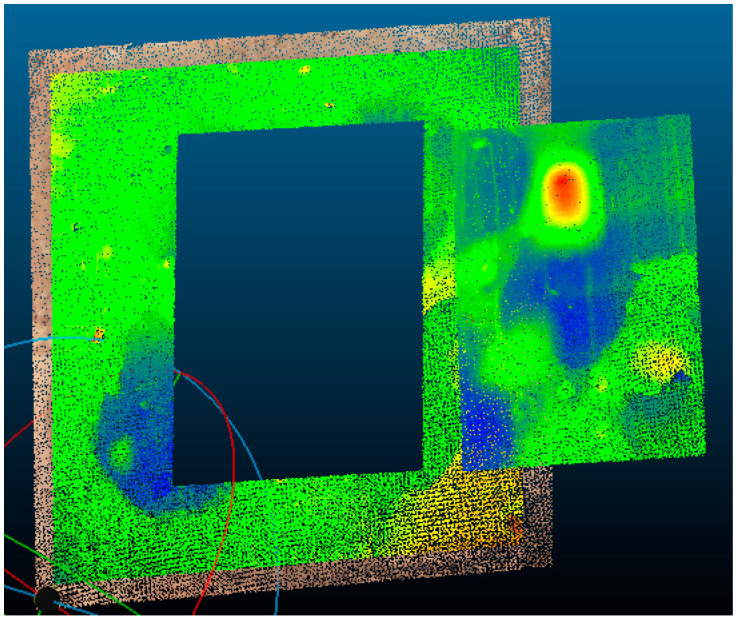
Extraction of the Cloud A region of interest.

To this end, the delimitation and extraction of a region of common interest to the three point clouds were carried out for all clouds, as shown in Figure 14 for point Cloud A.

**Figure 15:**
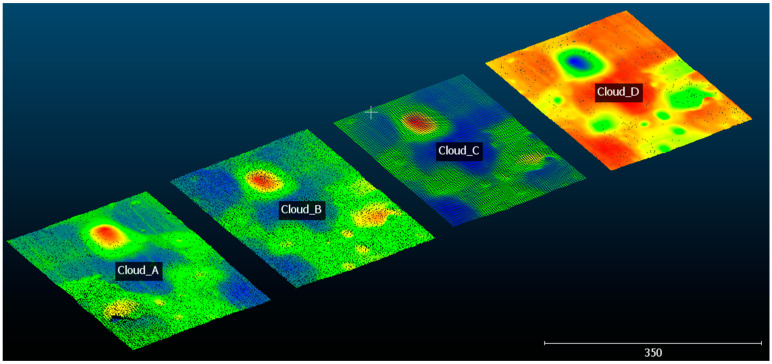
Point clouds A, B, C, and D.

In Figure 15, it possible to see the regions of interest extracted from the point clouds that correspond to Clouds A, B, C, and D.

To achieve the results presented in Figure 15, an alignment process was carried out between clouds A, B, C, and D using the Interactive Closest Point (ICP) algorithm proposed by Besl et al. [39] and Zhang [40]. During the treatment of the point clouds, the ICP algorithm allowed the identification of non-coincident regions between the clouds, which were eliminated. This rough alignment was necessary to extract the same region of interest of all clouds and consider it to estimate the reference planes. The application of this algorithm is quite widespread today to perform alignments between point clouds, and it proves to be quite reliable for high-accuracy applications, as proposed by Xue et al. [41] and Senin et al. [42], who applied the ICP algorithm to align point clouds with different point densities, as is the case in this work.

**Figure 16:**
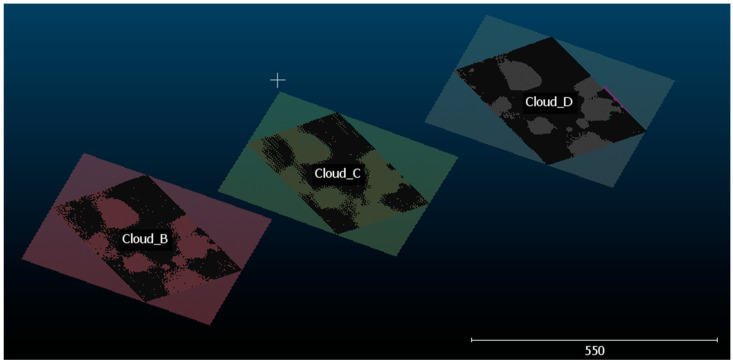
Random Sample Consensus RANSAC applied to clouds B, C, and D, and reference planes for each data set.

After cloud segmentation, an algorithm based on the Random Sample Consensus (RANSAC) method proposed by Schnabel et al. [43] and implemented in the CloudCompare software was applied to detect an existing planar region in each cloud (Figure 16). The criterion used to estimate planes was mainly the predefined angle α < 25°, which considers the angle between the normal of the candidate plane and the normal of the plane, initially defined by the three points chosen randomly at each iteration of the adjustment process of the best plane [43]. This plane was used as a reference plane and to determine a scalar field in each point cloud and finally measure the maximum depth for each trauma.

Concerning this, RANSAC was selected for the reasons described in [44], mainly due to the robustness with which it can handle outliers and clouds of different sizes. Considering that our data only have smoothed sloped surfaces, RANSAC has good behavior when applied to this data set to recognize planes.

### 5.6. Estimation of Penetration Depths Using Reference Planes

**Figure 17:**
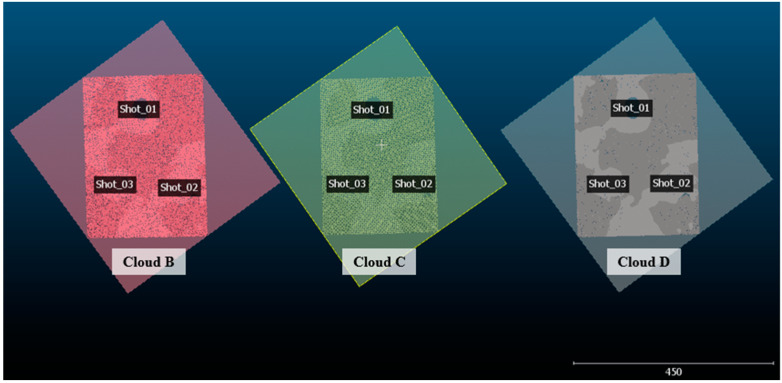
Trauma regions identified in clouds B, C, and D.

Once the reference planes were successfully estimated for each point cloud, it was possible to recognize the trauma regions in each set of points (Figure 17). These regions were manually segmented and had their depth measured using the reference plane previously defined.

Finally, to compare the performance of the techniques used, the maximum depth values measured were extracted from the scalar fields computed for each cloud and compared.

## 6. Results and Discussions

**Figure 18:**
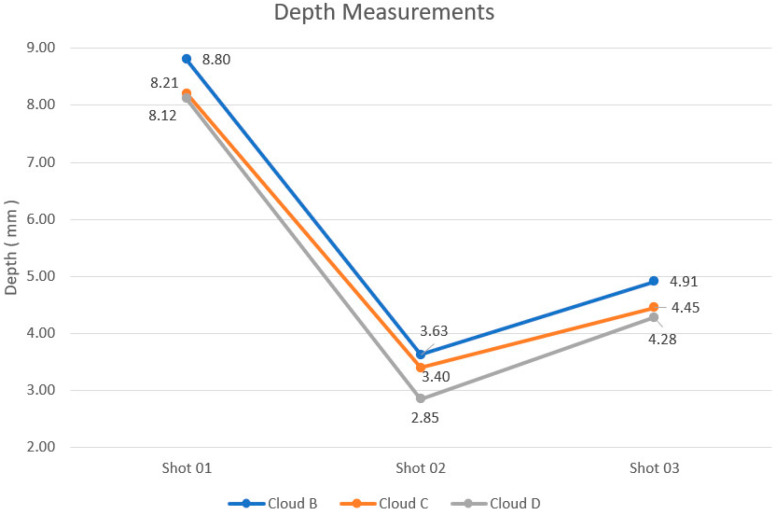
Graphical representation of the measurements taken considering each point cloud and also the deformations of the traumas.

With the application of the procedure described in the previous section, it was possible to obtain the information described in Table 2 and shown in Figure 18. The table and the figure contain information on the measurement results, using the FAROARM point cloud measurements as a reference.

As expected, due to the different measuring principles and instrument performances, each technique generated different results for the depth of the same shot. Comparing the results of Clouds B and C in Table 2 and Figure 18, it was found that the values of Cloud C were lower in their entirety, characterizing a trend of the technique of 3D reconstruction by stereo images to yield depth values below those observed for the 3D Handheld Scanner.

With the values obtained for each depth measurement, the differences between the depths of point clouds B and C in relation to the point cloud taken as reference (Cloud D) were calculated. The difference values obtained are presented in Table 3, and also the average of the errors, the standard deviation, and the type “A” uncertainty.

Lower differences were obtained for the 3D reconstruction technique, and it is possible to assume that these results are very close to the actual values. Although the standard deviation and type “A” uncertainty are smaller for the handheld scanner, as seen in Figure 18 and Table 3, the average error was about three times higher than that observed for the 3D reconstruction technique, which demonstrates that this technique can be applied in this measurement process with satisfactory results and even better than using the handheld 3D scanner. The maximum difference observed was 0.78 mm for the handheld 3D Scanner, below the 1 mm resolution defined by the NIJ 0101.04 [3] standard, allowing the application of both techniques in the measurement process considered. Xia et al. [28] obtained very similar results when comparing a handheld scanner and a static scanner. Although the results were for the comparison between the two scanners, it is possible to establish common points between the present work and the one presented by Xia et al. In both works, the best results were obtained by static acquisition techniques.

In practical terms, despite the larger associated errors, the handheld 3D scanner is more suitable for the application of interest, considering that it still meets the standard requirements, mainly due to its ease of use. Even though the 3D reconstruction technique has shown better results than the handheld 3D scanner at a relatively low associated cost, its application is more challenging because of the configuration for acquiring images of this type of test in question. On the other hand, the measuring arm stands out as the best solution; however, the cost involved—of up to five to six times more than the handheld unit—would make its application unfeasible.

## 7. Conclusions and Future Work

Intending to evaluate the applicability of the 3D reconstruction technique in measuring trauma in body armor tests, the present work sought to compare different 3D scanning techniques, comparing the point clouds obtained from the same object of interest.

It was possible to compare the results using the RANSAC methodology, and it was found that, for the experiments carried out, although the variability is higher for the reconstruction method compared to digitization using the scanner, the 3D reconstruction using stereo images showed values closer to the real values for all measurements performed in the regions of interest. It was observed that the average of the observed errors was also considerably lower.

The results using 3D reconstruction obtained satisfactory results for the comparison in question. Some reasons may explain this good performance:

- As the 3D reconstruction technique is based on the use of stereo images of a given object, and considering that the surface presented relatively small deformations when compared to others commonly observed in ballistic tests, the focal distance for capturing the images allowed good depth estimates. The observed fact also explains the ease in establishing reference plans using the RANSAC methodology. Possibly, the result would be different with very deformed surfaces.
- For 3D Scanner data acquisition, the operator must do some small movements around the object of interest, and these movements can be a source of error. In the 3D reconstruction process, the acquisition is made statically.

Considering that the results of 3D reconstruction were satisfactory in the study in question, further investigations would be necessary to understand the maximum depth values to be measured by this technique without losing the accuracy required for compliance with the current standards.

Three-dimensional scanning instruments used for the intended application bring numerous benefits, such as the possibility of the automated measurement of traumas through the recognition of shapes and algorithms, the elimination or reducing of the operator’s influence on measurements, and the possibility of archiving the scans as part of the process.

Additionally, it was observed that, despite the specific software employed in this study being an effective tool, it would be interesting to develop a specific computational implementation for this purpose. The application of an automated signal processing algorithm would be simpler and would reduce the time spent in the entire process.

Considering that the treatment of point clouds was extremely relevant for the extraction of information of interest, special attention should be given to the process in order to find the best methods for establishing reference planes, removing outliers, and identifying points of larger depth in trauma regions.

Bearing in mind that some factors, such as the luminosity, distribution and quantity of speckles, and focal length, can influence the results obtained, further experimental investigations would be necessary to analyze how each of these can influence the measurement.

The use of the feature point histogram technique, as identified in the works [41,45], and some variations [46] that use histograms to identify objects in the clouds associated with machine learning techniques are promising, since it would be possible to automatically recognize trauma regions.

Finally, for future research, the possibility of using mathematical tools and methodologies as proposed by Pandey et al. [47] can be applied, which would make use of the information present in point clouds to perform the alignment, taking as reference the dominant planes present in each set. Projection Pursuit [48] or Principal Component Analysis (PCA) [49] can be used to find the dominant plane in point clouds originated from trauma scans, as was explored in the present study, and can be used as a reference for depth measurements.

It is also believed that the tests performed can be repeated considering a larger number of points of interest. Another possibility is a flatness study for different digitization techniques. However, for this, it would be necessary to scan a calibrated surface plate, as it would thus be possible to observe possible “deformations” between the techniques, which evidently would demand more experimental work.

## Figures and Tables

**Table 1:** Levels established by NIJ 0101.04 [3].

Level	Ammunition	Speed
I	0.22 LR	329 ± 9 m/s
	0.380 ACP	322 ± 9 m/s
IIA	9 mm	341 ± 9 m/s
	0.40 S & W	322 ± 9 m/s
II	9 mm	367 ± 9 m/s
	0.357 Mag	436 ± 9 m/s
IIIA	9 mm	436 ± 9 m/s
	0.44 Mag	436 ± 9 m/s
III	7.62 mm NATO	847 ± 9 m/s
IV	0.30 M2 AP	878 ± 9 m/s

**Table 2:** Depth estimates from the measurements made with a 3D handheld Scanner, 3D Reconstruction, and FAROARM 3D Scanner.

Clouds	Depth Estimates (mm)		
	Shot 01	Shot 02	Shot 03
B (Handheld Scanner—Milled Plate)	8.80	3.63	4.91
C (3D Reconstruction—Milled Plate)	8.21	3.40	4.45
D (FARO Arm Scanner—Milled Plate)	8.12	2.85	4.28

**Table 3:** Differences in mm for the depth estimates made using cloud D as a reference.

Clouds	Differences in mm				Values in mm	
	Shot 01	Shot 02	Shot 03	Average	Standard Deviation	Uncertainty
Cloud B–Cloud D	0.68	0.78	0.63	0.70	0.08	0.04
Cloud C–Cloud D	0.09	0.55	0.17	0.27	0.25	0.14
